# Alpha‐Oscillatory Current Application Impacts Prospective Remembering Through Strategic Monitoring

**DOI:** 10.1111/psyp.70024

**Published:** 2025-03-16

**Authors:** Bruno de Matos Mansur, Viviana Villafane Barraza, Angela Voegtle, Christoph Reichert, Slawomir J. Nasuto, Catherine M. Sweeney‐Reed

**Affiliations:** ^1^ Department of Neurology, Neurocybernetics and Rehabilitation Otto von Guericke University Magdeburg Germany; ^2^ Department of Behavioral Neurology Leibniz Institute for Neurobiology Magdeburg Germany; ^3^ Biomedical Sciences and Biomedical Engineering Division, School of Biological Sciences University of Reading Reading UK; ^4^ Center for Behavioral Brain Sciences Otto von Guericke University Magdeburg Germany

**Keywords:** delayed intention, EEG, future intention, prospective memory, source activation, strategic monitoring, tACS

## Abstract

Prospective memory (PM) is the ability to remember to execute future intentions. PM requires engagement of attentional networks, in which oscillatory activity in the alpha frequency range has been implicated. The left dorsolateral prefrontal cortex (DLPFC) and inferior parietal cortex are assumed to be engaged during PM tasks. We hypothesized that the selective application of transcranial alternating current stimulation (tACS) at alpha frequency to these areas can modulate PM‐associated event‐related potentials. Participants were assigned to alpha‐tACS, theta‐tACS, or Sham stimulation. They performed a working memory task (OGT), with a PM component, pre‐, during, and post‐stimulation. EEG was recorded post‐stimulation. Accuracy and reaction times (RTs) were computed. Following EEG source reconstruction of mean amplitude, source activity was contrasted between conditions in which performance was modulated by tACS using cluster‐based permutation tests. RTs were slower on introducing the PM task, consistent with strategic monitoring. PM accuracy improved in the alpha‐tACS group only. During PM trials, source activity in the posterior cingulate cortex (PCC) was lower following alpha‐tACS than after Sham stimulation. Source activity in the DLPFC following alpha‐tACS was lower during PM than in OGT trials following alpha‐tACS. Performance modulation through alpha‐tACS, and the lower DLPFC activity in PM than in OGT trials provide evidence of a role for alpha oscillations during strategic monitoring for a PM cue. Lower PCC activity in the alpha‐tACS than Sham group is consistent with facilitation of disengagement of the default mode network, supporting re‐direction of attention from the OGT to the PM task and task‐switching.

## Graphical abstract

Prospective memory (PM) performance and known neural correlates of PM were modulated through application of frontal and parietal transcranial alternating current stimulation at alpha frequency (alpha‐tACS). Alpha‐tACS improved PM accuracy. During PM trials, alpha‐tACS reduced early mean amplitude in the source reconstruction of the posterior cingulate cortex compared to a control group and reduced the late mean amplitude in the source reconstruction of the prefrontal cortex, comparing PM and working memory trials. The findings provide evidence for a role for alpha oscillations in PM processing.

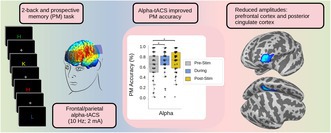

## 1 Introduction

Prospective memory refers to memory for future intentions and is essential for guiding our actions in daily life (Schacter and Addis 2007; West 2011; West and Krompinger 2005). Prospective memory impairment is among the most frequently reported memory failure complaints (Kliegel and Martin 2003) and can pose substantial challenges to daily activities, including medication compliance (Fish et al. 2010). Prospective memory deficits are seen in a range of conditions, including mild cognitive impairment (Costa et al. 2011), Parkinson's disease (Costa et al. 2012), and also in normal aging (Einstein and McDaniel 1990; West et al. 2003). Understanding the neural correlates of prospective memory has the potential to aid diagnosis and to inform neuromodulatory approaches to improving prospective memory.

Prospective memory evaluation requires a prospectively encoded intention to perform a particular action, a time delay until the action is performed, and an ongoing task that serves as a distraction, preventing participants from continuously thinking about the planned action (Einstein and McDaniel 1990). Prospective memory may be achieved through strategic monitoring of the environment for a prospective memory cue, spontaneous retrieval of a prospectively encoded intention, or a combination (Guynn 2003; McDaniel and Einstein 2007). Spontaneous retrieval may occur when the environmental cues naturally prompt the recall of the intended action. In contrast, individuals employing strategic monitoring actively allocate attention and mental resources toward noticing specific cues associated with the intended action. This intentional attention allocation to the prospective memory task can interfere with ongoing task performance (McDaniel and Einstein 2000, 2007; Smith 2003).

Attentional processing is a key component in achieving prospective memory through strategic monitoring. Cortical oscillatory brain activity in the alpha frequency range (8–12 Hz) has been shown to be modulated by attentional demand (Misselhorn et al. 2019) and by prospective memory processing (Martin et al. 2007). Neuroimaging studies have revealed engagement of frontal and parietal cortical regions in prospective memory (Cona et al. 2023). Changes in left dorsal frontal–parietal activity in the alpha frequency range have been reported when attention is externally directed to monitor the environment for prospective memory cues (Cona et al. 2020). In a study examining oscillatory activity in two different prospective memory tasks, designed to encourage different prospective memory strategies, modulation of alpha activity was only observed in the task variant that facilitated strategic monitoring, by employing a non‐salient prospective memory cue (Cona et al. 2020). Notably, alpha activity was reduced early over bilateral posterior regions and then later in left central and frontal regions. We recently observed an initial increase followed by reduction in alpha activity on prospective memory retrieval compared with ongoing task retrieval, both in parietal and frontal cortical regions (Villafane Barraza et al. 2023). This difference peaked in the parietal cortex, which has an established role in memory retrieval (Rugg et al. 2008; Watrous et al. 2013).

Synchronization of oscillations in brain electrical activity in slow oscillatory frequency ranges, such as alpha and theta (4–8 Hz), is thought to provide a mechanism by which remote brain areas communicate and to facilitate neural plasticity (Buzsáki and Draguhn 2004; Lachaux et al. 1999; Varela et al. 2001). Cognitive processes, such as those underlying working memory (WM), are considered to be underpinned by neural assemblies formed through transient synchronization of oscillatory activity in particular brain regions (Sarnthein et al. 1998). Previous studies have shown that these processes can be modulated through application of transcranial alternating current stimulation (tACS), providing causal investigation of modulatory neural bases associated with specific behavioral outcomes (Polanía et al. 2012; Röhner et al. 2018; Violante et al. 2017). In the present study, by applying tACS at an alpha frequency (alpha‐tACS), we investigated whether entrainment of alpha oscillatory activity in two brain areas deemed to be critical nodes in the brain networks underpinning prospective memory would modulate prospective memory performance and its established electrophysiological correlates. We investigated effects on prospective memory maintenance and retrieval by contrasting effects on performance of an ongoing task baseline with those when a prospective memory component is included in the task, and by comparing the effects of alpha‐tACS on prospective memory trials with two control conditions: theta‐tACS, to evaluate frequency specificity, and sham stimulation. Theta oscillations have a well‐established role in memory processing, and several studies indicate that theta‐tACS modulates associative memory and WM performance (Lang et al. 2019; Polanía et al. 2012; Röhner et al. 2018; Violante et al. 2017). Given the differential modulation of alpha and theta power, dependent on the prospective memory strategy employed (Cona et al. 2020), theta‐tACS was not predicted to modulate the strategic monitoring expected to be used in the task employed here.

Previous studies have shown that prospective memory can be modified by transcranial stimulation applied to frontal and parietal regions (Bisiacchi et al. 2011; Cona et al. 2017). These studies employed repetitive transcranial magnetic stimulation. However, studies applying single sessions of transcranial direct current stimulation (tDCS) to the left and/or right dorsolateral prefrontal cortex (DLPFC) have not shown any modulation of event‐related prospective memory (Aksu et al. 2024; Ellis et al. 2020; Rose et al. 2020). Here, we extend this work using transcranial alternating current stimulation, which enables modulation of oscillations at specific frequencies, and we also examine the impact on electrophysiological measures.

We used the 2‐back behavioral paradigm as the ongoing task, as it has been shown to have sufficient cognitive load to prevent continual prospective memory rehearsal (Möschl et al. 2019; West and Bowry 2005). Our choice of the n‐back task for the ongoing task was based on previous prospective memory studies using this ongoing task, with a different color used as the prospective memory cue and pressing a different response button as the prospectively encoded intention (West and Bowry 2005; Möschl et al. 2019; Flanagan et al. 2024). Specifically, in choosing a demanding ongoing task, we aimed to test prospective memory rather than dual performance of parallel tasks. While a prospective memory task may be deemed technically to be a type of dual task paradigm (Lewis‐Peacock et al. 2016), testing prospective memory is considered to require several particular paradigm features: (1) an ongoing task with a high enough cognitive load to stop participants from continuously actively rehearsing the prospective memory part of the paradigm (Einstein and McDaniel 1990; Guynn 2003; West 2008); (2) a delay between forming the prospective intention and carrying it out (Smith 2003); (3) lower frequency prospective memory than ongoing task items (Wilson et al. 2013); (4) task switching, with inhibition of the ongoing task response (Bisiacchi et al. 2009; McDaniel and Einstein 2007).

Event‐related potentials (ERPs) reflect alterations in the activity of large numbers of neurons and have been found to be related to changes in oscillatory power at different frequencies (Makeig et al. 2002; Buzsaki 2006). While ERPs comprise summed postsynaptic potentials, reflecting multiple neuronal processes, their measurement provides a robust starting point for evaluating whether brain activity is altered by application of external, transcranial currents. Scalp maps of ERPs comprise contributions from multiple cortical sources, and evidence from visual potentials suggests that ERPs may arise through stimulus‐induced phase resetting of ongoing field potential oscillations (Makeig et al. 2002). The aim in applying source ERP analyses in the current study was to identify activity modulation in task‐relevant cortical areas. Multiple cortical alpha rhythms have been observed, which have different yet overlapping scalp distributions and are associated with separate cognitive processes (Lutzenberger 1997; Andrew and Pfurtscheller 1997). We examined the source reconstruction of the mean amplitude of event‐related source activity to include contributions from modulation of rhythms in multiple frequencies, including the alpha and theta ranges, to evaluate whether tACS can alter processing associated with any induced prospective memory‐related behavioral changes. In particular, online (during stimulation) changes in oscillatory activity have the potential to lead to offline (post‐stimulation) changes that may be reflected in different rhythms to those modulated.

To evaluate whether any stimulation‐associated behavioral effects were accompanied by prospective memory‐relevant neuromodulation, we selected two time windows based on ERP studies: An early window to examine effects on the N300 and frontal positivity (200–400 ms post‐stimulus) and a late window to assess a potential impact on prospective positivity (600–800 ms post stimulus). The N300, frontal positivity, and prospective positivity have been specifically related to prospective memory retrieval (Gonen‐Yaacovi and Burgess 2012; West 2011). The N300 occurs in a parieto‐occipital region between 200 and 400 ms after prospective memory cue presentation (Cruz et al. 2016; West 2011; Wilson et al. 2013). It is associated with prospective memory cue detection (Bisiacchi et al. 2011) and reflects processing differences between ongoing task and prospective memory trials. Frontal positivity occurs over midline frontal brain regions, around 200 ms post‐stimulus, and may last until 800 ms after stimulus onset (Gonen‐Yaacovi and Burgess 2012; West 2008; Wilson et al. 2013). Frontal positivity has been proposed to be indicative of cognitive processes involved in redirecting or disengaging attention from ongoing activities to focus on fulfilling the requirements of the prospective memory task (West 2004; Wilson et al. 2013). Finally, prospective positivity may occur in central parietal and occipital regions between 400 and 1200 ms after prospective memory cue presentation (Cruz et al. 2016; Gonen‐Yaacovi and Burgess 2012). It is considered to reflect the mental representation of currently performed or planned tasks, prospective memory cue evaluation, and task switching (West 2011; Wilson et al. 2013). Meta‐analyses point to consistent activation of several brain structures and networks during prospective memory processing (Cona et al. 2015, 2023). Consistent activation in the anterior prefrontal cortex (aPFC) and areas within the dorsal fronto‐parietal network, including the DLPFC and the posterior parietal cortex, has been observed during prospective memory maintenance (Cona et al. 2015). Parietal regions are considered to play a role in the strategic redirection of attention towards external prospective memory cues and the content of future intentions, which are represented by frontal regions (Burgess et al. 2007; Cona et al. 2015, 2023). The salience network, comprising the insula and anterior cingulate cortex (ACC), is deemed to operate as a switch in prospective memory tasks, directing attention toward internal and external cues and communicating with other networks, including the default mode network (Cona et al. 2023). Within the latter, the lateral aPFC, on receiving information regarding a conflict between two goals from the ACC, regulates information favoring execution of the prospective memory intention, whereas the posterior cingulate cortex (PCC) and ventral frontoparietal regions facilitate spontaneous attention processes triggered by the prospective memory stimulus, redirecting attention toward the memory representation of the future intention (Cona et al. 2015). Given the engagement of multiple brain regions and networks in prospective memory, with the potential for network effects of stimulation, we took a whole brain approach to evaluating the impact of tACS during prospective memory. We also examined the after‐effects of stimulation on alpha and theta oscillations.

Based on the engagement of the inferior parietal cortex (IPC) and the DLPFC during prospective memory tasks (Cona et al. 2015), and evidence that transcranial stimulation of these areas can modulate prospective memory (Bisiacchi et al. 2011; Cona et al. 2015), whereas tDCS shows no effect (Aksu et al. 2024; Ellis et al. 2020; Rose et al. 2020), we hypothesized that tACS at a task‐related frequency applied to the left IPC and DLPFC can enhance prospective memory performance by modulating prospective memory processing and its well‐established electrophysiological correlates in a strategic monitoring task. We applied current flow modeling to determine electrode placement to optimize current flow to these regions (Thielscher et al. 2015).

## 2 Materials and Methods

### 2.1 Participants

A power analysis was applied for sample size calculation using G*Power, based on the findings of a study in which tACS was applied in a cross‐over design during an attentional task, and between‐group differences in ERP amplitude were examined (Dallmer‐Zerbe et al. 2020). Based on an alpha threshold of 0.05 and power of 80%, 13 participants would be required per group to observe the difference. To account for any data loss due to technical difficulties during recording or participants being unable to perform the task correctly, we aimed to recruit 20 participants per group. Healthy, young (age 21–35), right‐handed (according to the Edinburgh Handedness Inventory) participants (*N* = 60) were recruited through local advertisement at the Otto von Guericke University Magdeburg. All participants were required to be fluent speakers of German or English, with corrected or normal vision, and to have no metal implants or history of psychiatric or neurological disorders. Twenty participants were randomly allocated to each stimulation group (alpha‐tACS, theta‐tACS, and Sham). All participants performed the KAI short‐form intelligence test, based on WM capacity (Lehrl et al. 1992). The participants were informed of their right to interrupt or terminate the experiment at any time without providing a reason. All participants provided written informed consent before inclusion in the study, in accordance with the Declaration of Helsinki. Ethical approval for the study was obtained from the Local Ethics Committee of the University Hospital Magdeburg (16/20).

### 2.2 Study Design

**FIGURE 1:**
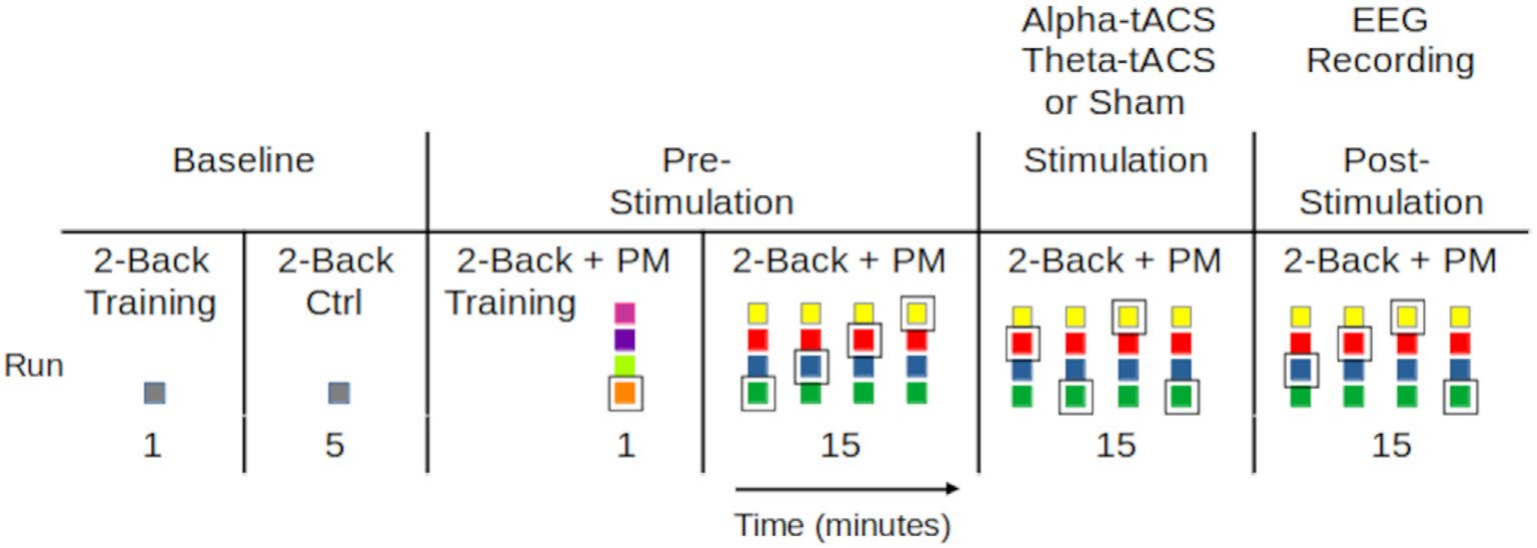
Timeline of the experiment. The Baseline consisted of one training run and one run of only the 2‐back task. Directly after, the three main blocks (pre‐, during, and post‐stimulation) followed, with four runs each. Colored rectangles represent the four possible letter colors, one of which was assigned as the prospective memory cue (surrounded here in a rectangular border) for each new run.

The experiment comprised four main blocks. The Baseline block consisted of a 2‐back task. The 2‐back task with an embedded prospective memory component was then implemented in the three main blocks: Pre‐, During, and Post‐stimulation (Figure 1). Stimulation was applied according to group allocation (alpha‐tACS, theta‐tACS, or Sham). Post‐stimulation, EEG data were recorded during task performance.

### 2.3 Stimulus Material: 2‐Back Plus Prospective Memory Task

**FIGURE 2:**
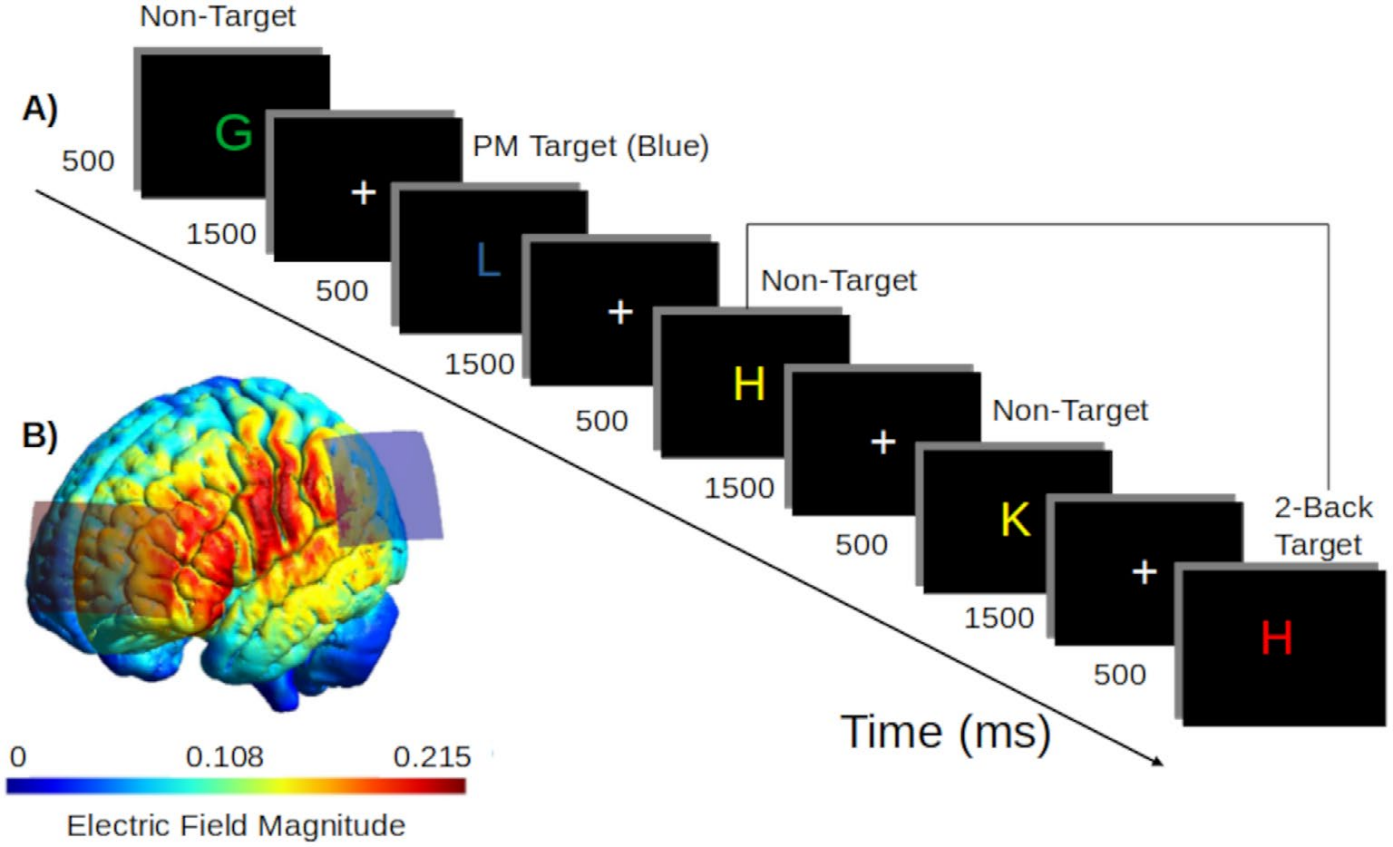
(A) 2‐back working memory task with embedded prospective memory (PM) task. In this example, the prospective memory cue was the color blue. Responding to the prospective memory cue was prioritized over the ongoing task. The next letters H (yellow) and K (yellow) were both nontargets, followed by H (red), a target of the ongoing task. (B) Simulated electric field magnitude (V/m^2^) for the transcranial alternating current stimulation montage.

The 2‐back task, a well‐established WM task (Kirchner 1958; Pesonen et al. 2007; Röhner et al. 2018; Voegtle et al. 2022), was used as the ongoing task. Uppercase letters (21 consonants ranging from ‘B’ to ‘Z’), in one of four colors (green, blue, red, and yellow), were displayed in a pseudorandom order in the center of a black screen for 500 ms, followed by a fixation cross for 1500 ms (1.5 cm in size, at a distance of 85 cm with a visual angle of 1.0°; Figure 2A). Three keyboard buttons were used. Participants were instructed to press a button with either the right index finger or the right ring finger (counterbalanced across participants) every time the letter displayed matched the letter presented two trials before (target). If the letter was not the same (non‐target), they were required to press the other button. In the prospective memory task, participants were told instead to press a button with the right middle finger if the letter displayed had a specific color, prioritizing the prospective memory response instead of responding to the ongoing task (prospective memory target). Inhibiting the ongoing task and task‐switching are considered to be a requirement for assessing prospective memory, as opposed to asking participants to respond first to the ongoing task before the prospective memory, which constitutes a dual‐task paradigm (Bisiacchi et al. 2009). The specified color for the prospective memory cue was assigned four times per block.

Each prospective memory block comprised four runs of 3.7 min (113 trials per run), and at the beginning of each run, a different prospective memory cue, which could be either green, blue, red, or yellow, was assigned. The number of possible correct responses (potential ongoing task hits = 34; ~30%; potential correct rejections = 66; ~59%; potential prospective memory hits = 13; 11%) was chosen based on previous 2‐back and prospective memory studies. To avoid continuous active maintenance of the prospective memory task, prospective memory trials should be around 10% (West 2008; Wilson et al. 2013). We used 11% for numerical reasons.

The Baseline block comprised one run of 5 min (137 trials; potential ongoing task hits = 45; ~32%; potential correct rejections = 92; ~68%). The accuracy and reaction times (RTs) of responses to prospective memory stimuli were used to index prospective memory performance. The task was presented using Presentation Software (Version 18.2 Build 02.18.16).

### 2.4 Current Modeling Simulation and Optimization

#### 2.4.1 Current Modeling Simulation

Electric field modeling of the brain was performed using the SimNibs 3.2 toolbox, which is suitable for generating tACS simulations (Saturnino et al. 2019). First, a leadfield was calculated using the nonlinear, high‐resolution (0.5 mm^3^) ICBM152 2009b template, based on a 10–20 system. The electric field caused by each electrode individually was simulated, and then a 3D‐rendering finite element head mesh was generated that could be used to determine the optimal electrode placement and stimulation parameters for targeting the specific brain regions (Saturnino et al. 2019).

#### 2.4.2 Current Modeling Optimization

The optimization of the electrode location was performed to maximize the stimulation intensity in the left DLPFC (MNI coordinates: −38, 30, 40) and in the left IPC (MNI coordinates: −46, −34, 52), within a radius of 2 cm. Maximum total current was 2 mA, maximum individual current was set to 1 mA, and target intensity was set to 100 and −100 V/m^2^. The optimization indicated locations, labeled according to the 10–20 EEG system. AF3 (0.08 V/m^2^; average angle across target: 49.1°) corresponds with the left DLPFC (BA46) and CP5 (0.09 V/m^2^, average angle across target: 26.0°) corresponds with the left IPC (BA40) as the most effective pair of stimulation sites for the tACS montage (Figure 2B).

### 2.5 Transcranial Alternating Current Stimulation

TACS was then administered using a NeuroConn DC‐Stimulator Plus device (serial 2049 Version 4.3.00.17) with two rubber electrodes (5 cm × 7 cm) inserted into saline‐soaked sponges (0.09%) to ensure optimal contact with the scalp and reduce impedance. The electrodes were placed under an EEG cap at the determined electrode positions, AF3 and CP5. Stimulation was applied at 6 Hz (theta‐tACS) or 10 Hz (alpha‐tACS) for 15 min, with peak‐to‐peak current intensity at 2 mA (between −1 mA and 1 mA) (Nitsche et al. 2003; Polanía et al. 2012) for the active stimulation groups. Current intensity was gradually ramped up and down for 15 s at the beginning and end of the stimulation. The sham group was stimulated at 10 Hz and only during the ramping process to ensure that all study participants were blinded to stimulation conditions (Gandiga et al. 2006). The impedance between the stimulation electrodes was kept under 20 kΩ.

### 2.6 Electrophysiological Recording and Preprocessing

After stimulation, brain activity was recorded with 64 actiCap electrodes (Brain Products, Gilching, Germany) using Brain Vision Recorder version 1.20.0001 (Brain Products, Gilching, Germany) at a sampling rate of 500 Hz in a shielded chamber. The reference electrode was placed at FCz, the ground electrode at AFz, and impedances were kept below 20 kΩ. Data analysis was conducted using MNE software version 1.2.1 (Gramfort 2013; Larson et al. 2022) running on Python version 3.10.6. Raw data were filtered between 0.1 and 48 Hz, and bad channels (three to four channels per participant, except for one participant in the theta‐tACS group, with nine) were visually identified, deleted, and then replaced by interpolation of weighted neighboring channels. Heartbeat, eye blink, and other artifacts were detected on visual inspection and corrected using fast independent component analysis (Hyvärinen and Oja 2000). ERPs were then determined by averaging across epochs, which ranged from 200 ms pre‐ to 2000 ms post‐stimulus, for each participant and condition, using the 200 ms prestimulus activity for baseline correction. Only epochs with correct responses were included in the analyses.

#### 2.6.1 ERP Sensor‐Level Analysis

Peak amplitude ERP analysis was conducted, focusing on predefined regions of interest (ROIs) and time windows where prospective positivity (600–800 ms, electrode locations = [‘P3’, ‘Pz’, ‘P4’]), frontal positivity (250–400 ms, [‘Fz’, ‘AF3’, ‘AF4’, ‘AF7’, ‘AF8’]), and N300 (200–400 ms, [‘PO9’, ‘POz’, ‘PO10’]) have previously been identified. Peak amplitudes were extracted within the predefined time windows of interest and averaged over electrode locations within the ROIs. For visualization, these sensor‐level ERPs were averaged across participants for each condition.

#### 2.6.2 Source Reconstruction

A source reconstruction procedure was applied to the mean amplitudes to estimate the locations of activity sources from the sensor‐level ERP data, based on the recordings at all 64 electrode locations. The fsaverage standard magnetic resonance image template (Destrieux et al. 2010) was warped and aligned to the sensor position in a standard 10–20 coordinate frame, then used to compute the source space and forward operator. The source space mesh grid representing the potential origins of brain activity comprised 4098 source points per hemisphere, with 4.9 mm spacing between the grid points. Volume conduction was estimated using a three‐layered (skin, outer skull, and inner skull) boundary element model. The noise covariance matrix of the sensor data was estimated from the 200 ms prestimulus baseline time window. The noise covariance from each participant's data was then projected to the source space using dynamic statistical parametric mapping (Dale et al. 2000) to create the inverse operator, yielding one amplitude estimate per time and location in source space. One source estimate was created separately for each participant and condition.

#### 2.6.3 Power Analysis

Time–frequency analysis was performed using five‐cycle Morlet wavelets for the frequencies 1–48 Hz, in steps of 1 Hz, from −200 ms to 2000 ms, with a temporal resolution of 10 ms. For further processing, power estimates were normalized using a decibel transformation and baseline‐corrected, with the baseline defined as −200 ms to 0 ms prestimulus. Mean alpha (8–12 Hz) and theta (4–8 Hz) power was computed by averaging over the same ROIs and time of interest as used in the ERP analysis, separately for condition and participants. Power peak values were identified by finding the maximum power value across the specified ranges for each frequency band (alpha and theta). The analysis was implemented using the Fieldtrip toolbox (Oostenveld et al. 2011).

### 2.7 Statistical Analyses

We took a hypothesis‐driven approach to the statistical analyses, defining at the outset the contrasts of interest, as opposed to combining all possible contrasts in a single model, due to resource constraints limiting the total participant numbers (Lakens 2022). Bonferroni correction for multiple comparisons was implemented where applicable. Cohen's d was computed to quantify the effect size of differences between conditions. Confidence intervals (95%) were calculated for the mean differences, providing an estimate of the variability of the observed effects.

#### 2.7.1 Statistical Analysis: Behavioral

Statistical analysis was performed using Pingouin Statistics in Python 0.4.0 (Vallat 2018) (GNU General Public License v3.0). Behavioral performance was quantified according to accuracy and RT. Baseline performance during the ongoing task was compared between the stimulation groups using the Kruskal–Wallis test to ensure that they did not differ prior to intervention.

To evaluate whether the participants were strategically monitoring for a prospective memory cue, the potential impact of introducing the prospective memory task on ongoing task performance was examined by comparing ongoing task accuracy and RTs from baseline with pre‐stimulation, using a Wilcoxon signed‐rank test.

The potential influence of alpha‐ and theta‐tACS on prospective memory performance was then examined for each stimulation group by comparing prospective memory accuracy and RTs Pre‐, During, and Post‐stimulation. As not all data were normally distributed according to the Shapiro–Wilk test, non‐parametric Friedman tests for repeated measurements were applied, followed by Wilcoxon's signed‐rank tests, with a significance threshold of *p* < 0.05.

#### 2.7.2 Statistical Analysis: ERP Sensor‐Level Analysis

Dependent t tests were conducted to assess the differences in peak amplitudes in the ongoing task and prospective memory conditions after sham stimulation. Independent t‐tests were conducted to assess the differences in peak amplitudes between sham and alpha‐tACS stimulation in the ongoing task and in the prospective memory conditions.

#### 2.7.3 Statistical Analysis: ERP Source Estimates

Source activity was compared between prospective memory and ongoing task ERPs after alpha‐tACS, based on the behavioral difference in this contrast. We also performed a post hoc comparison between prospective memory trials after alpha‐tACS and after sham stimulation, in the same Early and Late time windows as for the sensor‐level ERP analysis. A spatio‐temporal cluster‐based permutation test (Maris and Oostenveld 2007) was performed to test for source estimate amplitude differences between groups (alpha‐tACS and Sham) and tasks (ongoing task, prospective memory). Initially, significant F‐values were defined according to spatiotemporally adjacent points differing at a *p* ≤ 0.01 cluster‐forming threshold. F‐values were then summed for each cluster. Significant clusters were identified by performing 1000 permutations of this procedure, randomly assigning data to the two groups, and the size of the largest cluster for each permutation was used to build the null distribution, against which the actual data were compared to determine the *p*‐value. A standard threshold of *p* ≤ 0.05 was applied to reject the null hypothesis. To prevent extreme statistical values in regions with low signal or high variance, a hat variance adjustment (Ridgway et al. 2012) with sigma = 0.001 was applied.

#### 2.7.4 Statistical Analysis: Power Analysis

Dependent t‐tests were conducted to assess the differences in mean peal alpha and theta power values in the ongoing task and prospective memory conditions after sham stimulation. Independent t‐tests were conducted to assess the differences in mean peak alpha and theta power values between sham and alpha‐tACS stimulation in the ongoing task and in the prospective memory conditions. Mean peak amplitudes were displayed in units of microvolts squared (μV^2^).

## 3 Results

Nine participants were excluded from the behavioral analysis: five did not understand or failed to perform one or more parts of the behavioral task, one was excluded due to technical problems during recording, and three did not attend, leaving 51 participants (age *M* = 27, SD = 3.76; 16 female), with 17 per stimulation group for analysis. Age, gender, and KAI scores did not differ between the stimulation groups.

### 3.1 Behavioral Findings

#### 3.1.1 Baseline

During the Baseline, neither ongoing task accuracy (*p* = 0.363) nor the ongoing task RT (*p* = 0.64) differed between the groups. However, Pre‐stimulation performance differed between the groups, precluding a between‐subject behavioral comparison. Within‐subject analyses were therefore performed, examining potential performance changes within each stimulation group.

#### 3.1.2 Effect on Ongoing Task of Introducing a Prospective Memory Task

**FIGURE 3:**
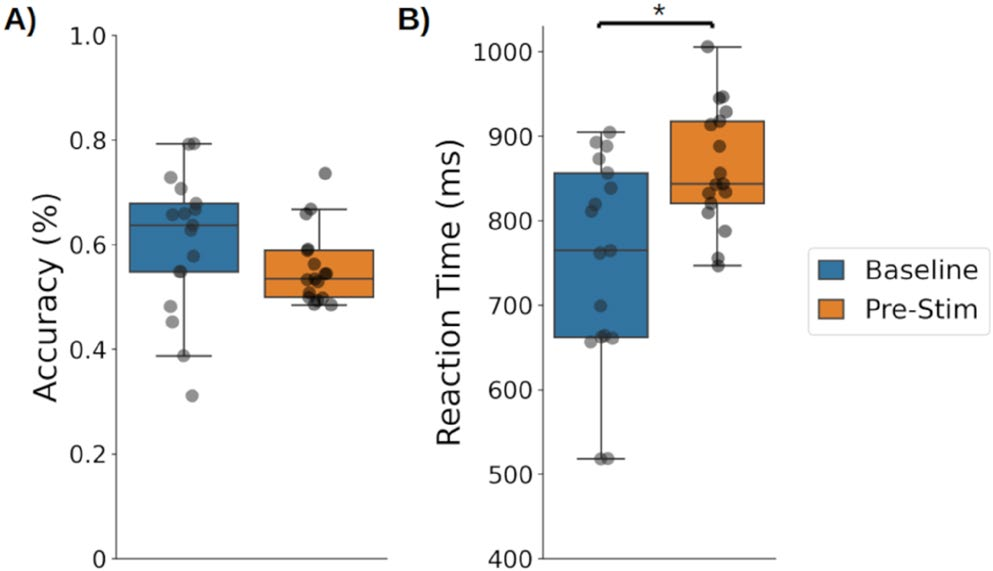
(A) Mean accuracy and (B) reaction time for the ongoing task trials during Baseline and Pre‐stimulation; Circles = individual mean values; * = *p* < 0.001.

Ongoing task RTs were slower after introducing the prospective memory task (Pre‐stimulation: 863 ms, 95% CI [0.83, 0.90]) than in the Baseline (752 ms, 95% CI [0.69, 0.81]; Wilcoxon's test: *z* = 11.0, *p* < 0.001, Cohen's *d* = 0.25; Figure 3B).

Ongoing task accuracy did not differ after introducing the prospective memory task (*p* = 0.21) (Figure 3A).

#### 3.1.3 Effects of Alpha‐ and Theta‐tACS on Prospective Memory

**FIGURE 4:**
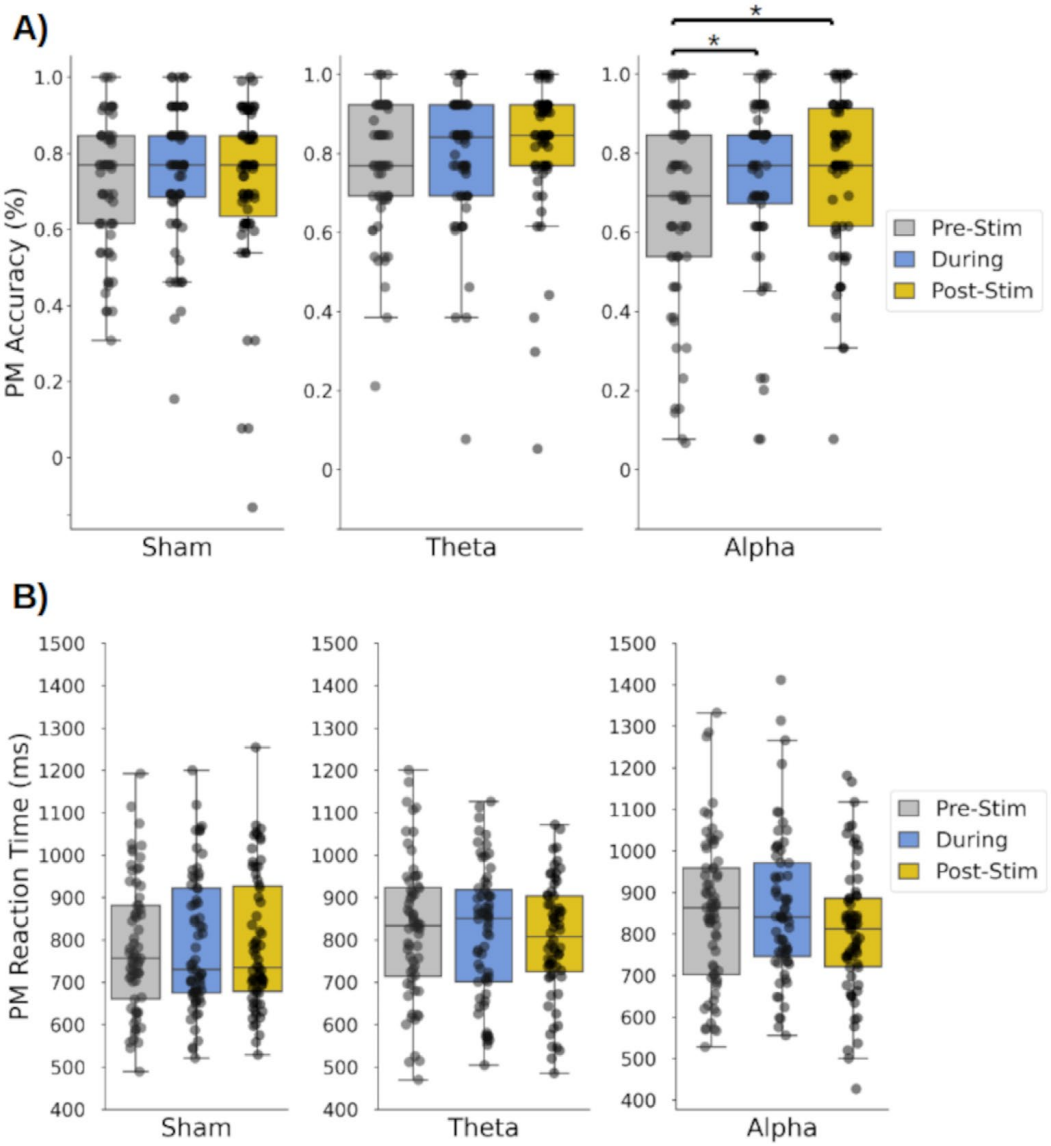
(A) Mean accuracy and (B) reaction time in the prospective memory trials Pre‐, During, and Post‐stimulation for each stimulation group (Sham, theta‐tACS, alpha‐tACS); Circles = individual mean values; * = significant difference at threshold *p* < 0.017 (after Bonferroni correction).

In the alpha‐tACS group, prospective memory accuracy differed between blocks (Friedman test: *χ* ^2^(2) = 6.17, *p* = 0.046; Figure 4A). Post hoc testing revealed greater prospective memory accuracy During (0.74, 95% CI [0.68, 0.79]) than Pre‐stimulation (0.68, 95% CI [0.62, 0.74]) (Wilcoxon's test: *z* = 587.5, *p* = 0.016, Cohen's *d* = 0.45) and also Post‐ (0.75, 95% CI [0.70, 0.80]) than Pre‐stimulation (Wilcoxon's test: *z* = 680.0, *p* = 0.010, Cohen's *d* = 0.45). Prospective memory RTs did not differ between blocks (*p* = 0.063; Figure 4B).

In the theta‐tACS and sham groups, neither prospective memory accuracy (theta‐tACS: *p* < 0.176; Sham: *p* < 0.336) nor prospective memory RTs (theta‐tACS: *p* = 0.252; Sham: *p* < 0.230) differed between blocks.

#### 3.1.4 Effects of Alpha‐ and Theta‐tACS on the Ongoing Task

**FIGURE 5:**
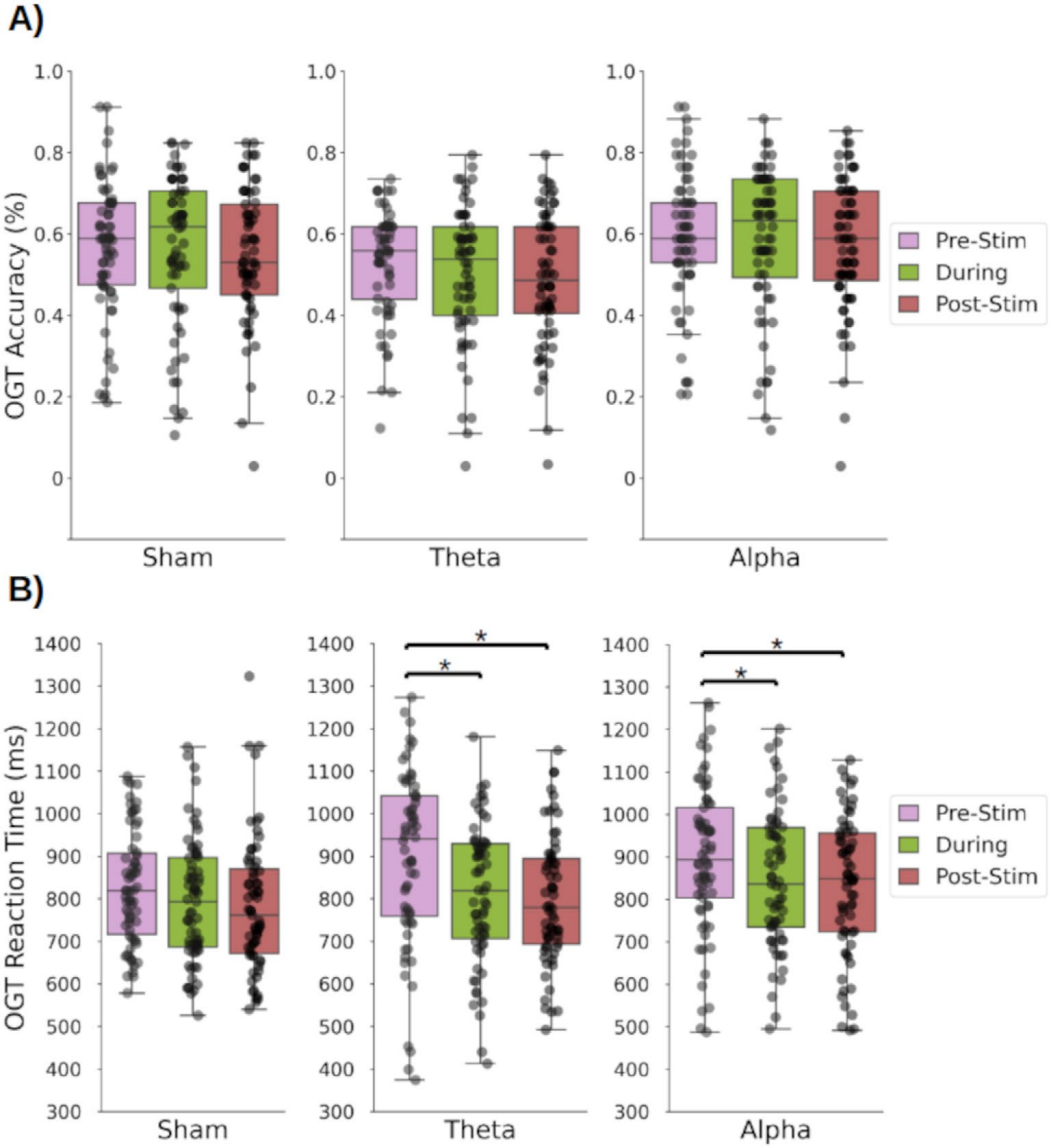
(A) Mean accuracy and (B) reaction time in the ongoing task (OGT) trials pre‐, during, and post‐stimulation for each stimulation group (Sham, theta‐tACS, alpha‐tACS); Circles = individual mean values; * = significant difference at threshold *p* < 0.017 (after Bonferroni correction).

In the alpha‐tACS group, ongoing task RTs differed between blocks (Friedman test: *χ* ^2^(2) = 19.35, *p* < 0.001; Figure 5B). Post hoc testing showed faster RTs During (846 ms, 95% CI [0.807, 0.886]) than Pre‐stimulation (900 ms, 95% CI [0.85, 0.94]) (Wilcoxon's test: *z* = 389.0, *p* = 0.001; Cohen's *d* = 0.60) and also Post‐ (830 ms, 95% CI [0.79, 0.87]) than Pre‐stimulation (Wilcoxon's test: *z* = 347.0, *p* = 0.001; Cohen's *d* = 0.60). Ongoing task accuracy did not differ between blocks (Figure 5A). Ongoing task RTs in the theta‐tACS group also differed between blocks (Friedman's test: *χ* ^2^(2) = 20.46, *p* < 0.001; Figure 5B). Post hoc testing showed that the theta‐tACS group had faster RTs During (810 ms, 95% CI [0.77, 0.85]) than Pre‐stimulation (900 ms, 95% CI [0.83, 0.96]) (Wilcoxon's test: *z* = 354.0, *p* = 0.001; Cohen's *d* = 0.64), and also Post‐(800 ms, 95% CI [0.76, 0.84]) than Pre‐stimulation (Wilcoxon's test: *z* = 346.0, *p* = 0.001; Cohen's *d* = 0.65). Ongoing task accuracy did not differ between blocks (Figure 5A).

### 3.2 Electrophysiological Findings

#### 3.2.1 ERP Analysis

Examining prospective memory accuracy, the alpha‐tACS group differed significantly from the other groups. We therefore examined electrophysiological differences between the alpha‐tACS and sham groups.

**FIGURE 6:**
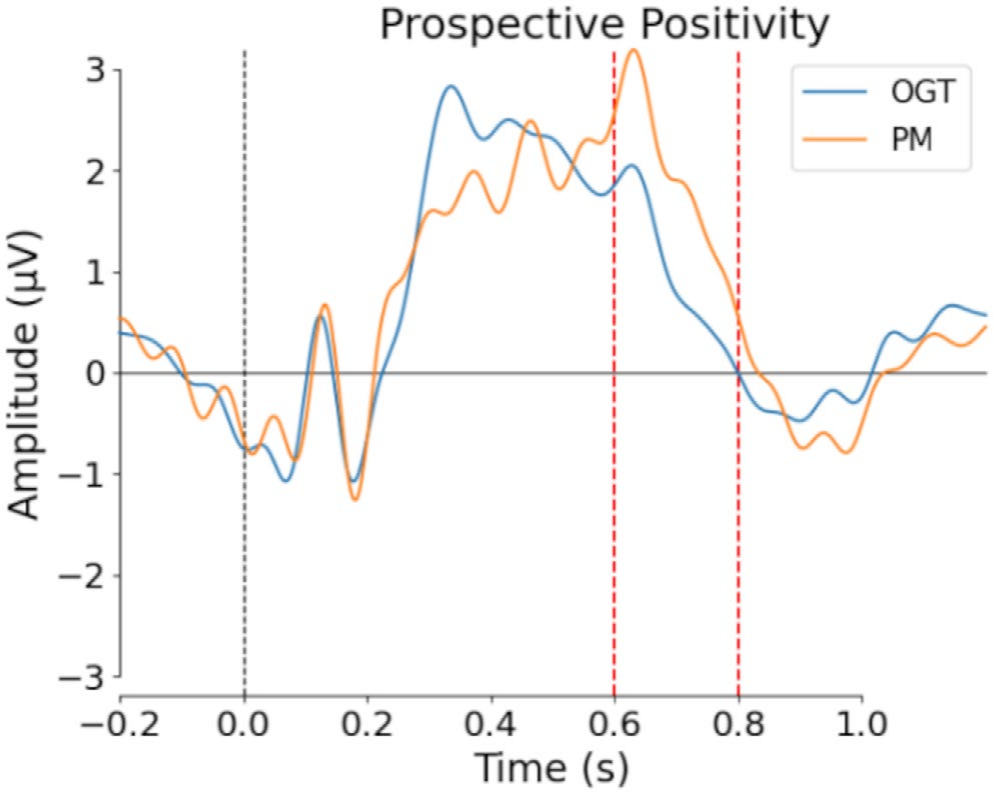
Grand‐average event‐related potentials following sham stimulation for a parietal region of interest (‘P3’, ‘Pz’, ‘P4’). The greater peak amplitude during prospective memory (PM) than ongoing task (OGT) trials in the time window between 600 to 800 ms is consistent with previously reported prospective positivity; Red dashed lines = significant difference at threshold *p* < 0.05.

After sham stimulation, we observed greater parietal positivity (prospective memory: 5.61 μV, ongoing task: 4.05 μV; 95% CI [3.50, 3.07]; *t*(15) = 2.18, *p* = 0.036; Cohen's *d* = 0.54) (Figure 6). We also observed a trend toward greater frontal positivity during prospective memory items (prospective memory: 1.51 μV, ongoing task: 0.39 μV; *p* = 0.12). No significant difference was seen for the N300 component (*p* = 0.52) or for the late frontal positivity (*p* = 0.96).

At the times and locations of established ERP correlates of prospective memory, no differences were observed between the alpha‐tACS and sham groups for prospective memory or for ongoing task trials. We therefore performed a source analysis on the basis that functional imaging studies have suggested the engagement of deeper cortical regions such as precuneus and ACC in prospective memory (Cona et al. 2015; Momennejad and Haynes 2013).

#### 3.2.2 Source Analysis

**FIGURE 7:**
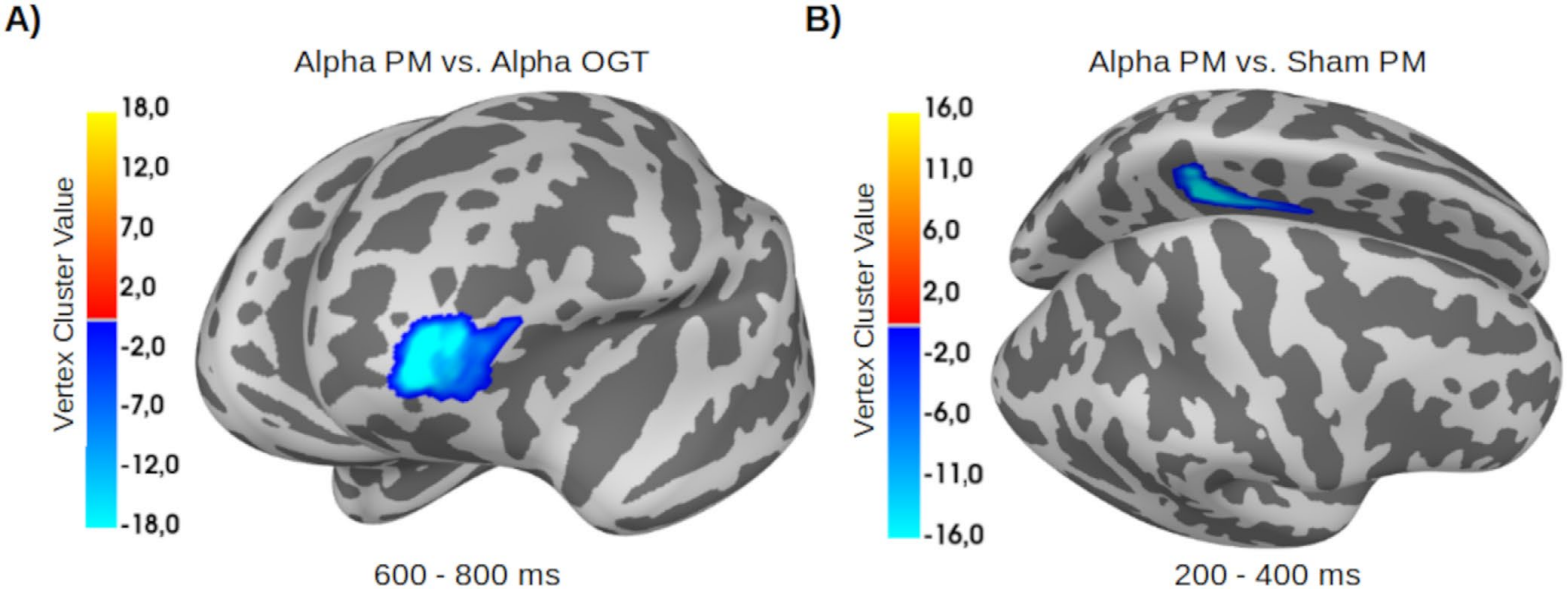
(A) Source reconstruction of mean amplitude differences between prospective memory (PM) and ongoing task (OGT) trials after alpha‐tACS at the border between the left anterior prefrontal cortex and dorsolateral prefrontal cortex. (B) Source reconstruction of mean amplitude differences between PM trials after alpha‐tACS and after sham stimulation in the left dorsal posterior cingulate cortex; The vertex cluster value indicates the temporal extent of the cluster at that vertex, measured in samples.

Following alpha‐tACS, a within‐subject analysis showed that the mean source amplitude in the prospective memory condition was lower than in the ongoing task condition in the Late time window over the border between the left DLPFC (BA 46) and the aPFC (BA 10) (cluster mass: 2009, *p* = 0.025, Cohen's *d* = 0.39). (Figure 7A). Source amplitudes in the Early time window did not differ.

After stimulation, a post hoc between‐subject analysis showed that source amplitudes were lower during prospective memory trials in the alpha‐tACS group than in the sham group in the Early time window. The difference was most pronounced near the left dorsal PCC (BA 31) (cluster mass: 1401, *p* = 0.030, Cohen's *d* = 2.46) (Figure 7B). Source amplitudes did not differ in the Late time window.

#### 3.2.3 Power Analysis

**FIGURE 8:**
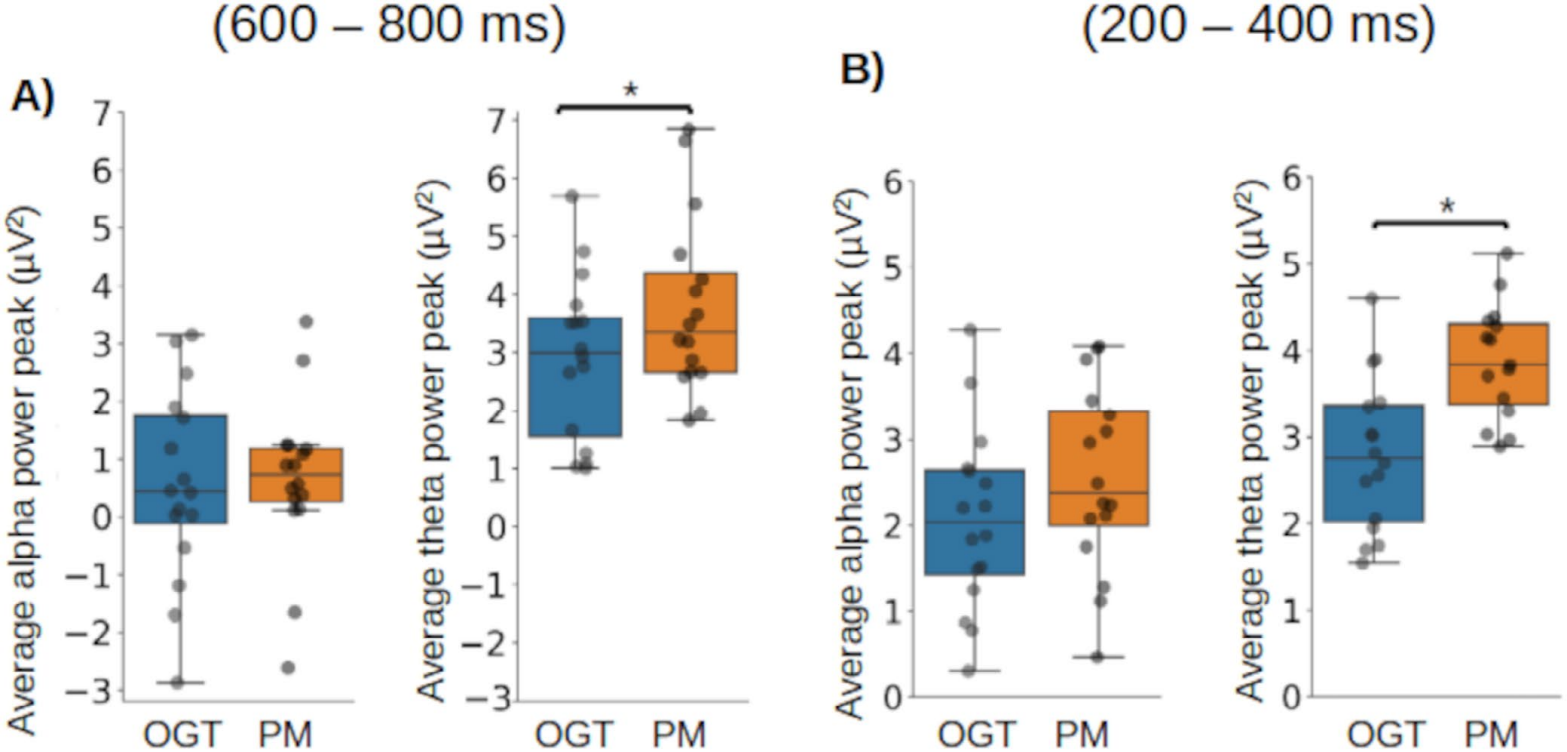
Mean alpha (left) and theta (right) power peak values after sham stimulation for prospective memory (PM) and ongoing task (OGT) trials at the time/location of ERP components reported in prospective memory. (A) For the time/location of prospective positivity, averaged over a time window from 600 to 800 ms and electrode locations ‘P3’, ‘Pz’, ‘P4’. (B) for the time/location of the N300, averaged over a time window from 200 to 400 ms and electrode locations ‘PO10’, ‘PO9’, ‘POz’; Circles = individual mean values; * = significant difference at threshold *p* < 0.05.

We then investigated whether oscillatory power differed between prospective memory and ongoing task items in the sham group at the times and locations where ERP differences have previously been reported. At the time and location of prospective positivity (late, parietal), theta, but not alpha, power was greater during prospective memory than the ongoing task (prospective memory: 3.22 μV^2^, ongoing task: 2.15 μV^2^; 95% CI [−1.70, −0.43]; *t*(16) = −3.60, *p* = 0.0026; Cohen's *d* = −0.90) (Figure 8A). At the time and location of the N300 (early, parietal), theta power was also greater during prospective memory than ongoing task trials (prospective memory: 3.87 μV^2^, ongoing task: 2.79 μV^2^; 95% CI [−1.61, −0.62]; *t*(15) = −4.85, *p* = 0.001; Cohen's *d* = −1.25) (Figure 8B), and alpha power showed a trend toward being greater during prospective memory (prospective memory: 2.54 μV^2^, ongoing task: 2.06 μV^2^; *p* = 0.088) (Figure 8B). At the time and location at which frontal positivity has previously been identified, no significant difference was seen between prospective memory and ongoing task trials.

We then investigated whether the impact of alpha‐tACS on prospective memory performance was accompanied by persistent oscillatory power differences at prospective memory‐relevant times/locations during strategic monitoring or on prospective memory retrieval by comparing alpha and theta power after stimulation in the alpha‐tACS ongoing task and sham groups.

**FIGURE 9:**
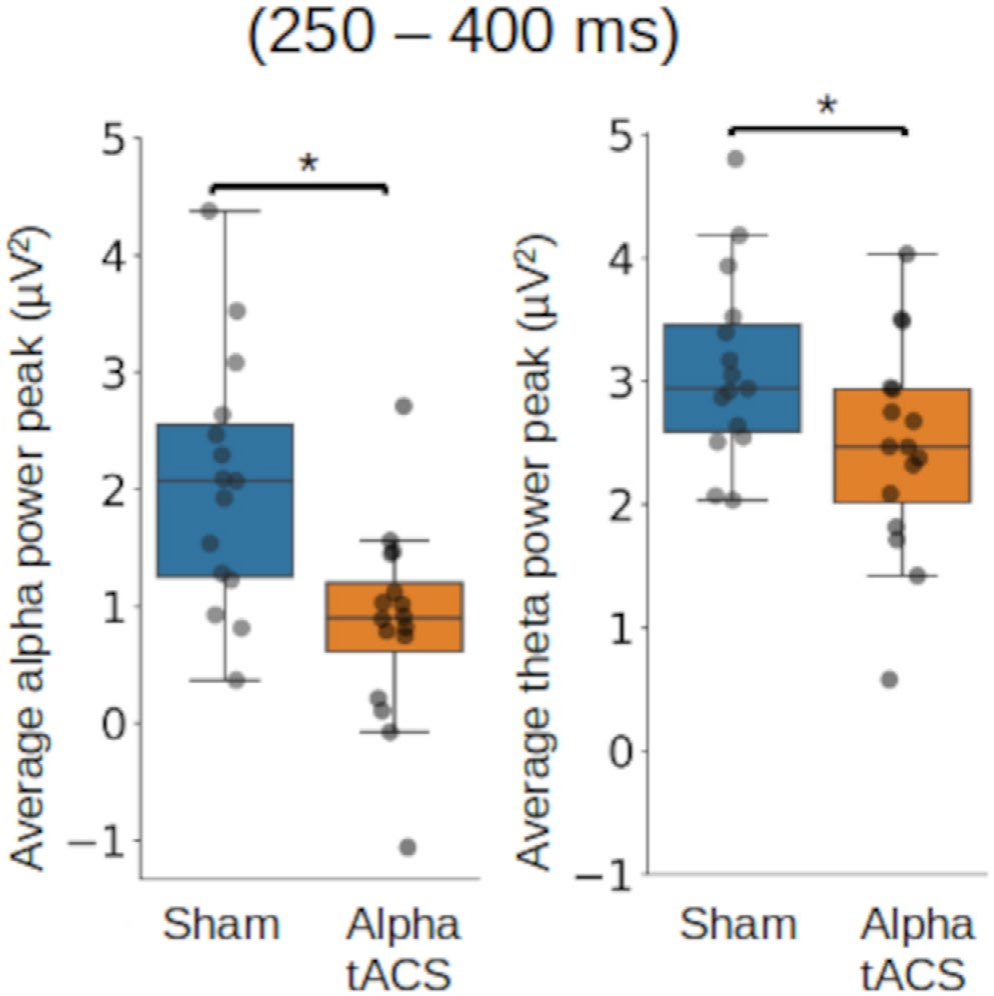
Mean alpha (left) and theta (right) power peak values following sham and alpha‐tACS for ongoing task trials at the time/location of frontal positivity, averaged over a time window from 250 to 400 ms and electrode locations ‘Fz’, ‘AF3’, ‘AF4’, ‘AF7’, ‘AF8’; Circles = individual mean values; * = significant difference at threshold *p* < 0.05.

During strategic monitoring (ongoing task trials while prospectively remembering), at the time and locations of frontal positivity (early, frontal), alpha power was lower in the alpha‐tACS group than in the sham group (alpha‐tACS: 0.86 μV^2^, Sham: 2.04 μV^2^; 95% CI [0.38, 1.97]; *t*(15) = 3.43, *p* = 0.0018; Cohen's *d* = 0.81; Figure 9),s and also theta power was lower in the alpha‐tACS than the sham group (alpha‐tACS: 2.47 μV^2^, Sham: 3.10 μV^2^; 95% CI [−0.12, 1.25]; *t*(15) = 2.16, *p* = 0.039; Cohen's *d* = 0.44; Figure 9). During the late frontal positivity, theta power showed a trend towards being lower in the alpha‐tACS than in the sham group (alpha‐tACS: 3.13 μV^2^, Sham: 4.42 μV^2^; *p* = 0.077). At the time and location at which N300 and parietal positivity have previously been identified, no significant differences were seen.

During prospective memory retrieval (prospective memory trials only), no differences in alpha or theta power were detected post‐stimulation at the times/locations of frontal positivity, the N300, or prospective positivity.

## 4 Discussion

We identified a specific improvement in prospective memory performance during alpha‐tACS, which persisted after stimulation. Prospective memory response accuracy was greater and could not be accounted for by a speed–accuracy trade‐off. RTs to ongoing task items were faster during and after than before alpha‐tACS, and while accuracy did not differ significantly, it did decrease, suggesting some possible speed–accuracy trade‐off. The effect was frequency‐specific, with no impact of theta‐tACS on prospective memory performance. These results are in agreement with studies demonstrating an improvement in prospective memory after repetitive transcranial magnetic stimulation (rTMS) (Bisiacchi et al. 2011; Cona et al. 2015), however, they stand in contrast to studies showing no effects of tDCS (Aksu et al. 2024; Ellis et al. 2020; Rose et al. 2020). We suggest that these contradicting results may reflect differences in electrode location as well as differences between these brain stimulation techniques. The rTMS study targeted the DLPFC and the posterior parietal cortex by stimulating the electrode locations F3, F4, P3, and P4 (Bisiacchi et al. 2011). Although the tDCS studies stimulated over similar electrode locations, rTMS has a greater modulation potency and probability of stimulating the targeted area than tDCS (Zhang et al. 2024). Based on these previous findings, we performed a simulation/optimization of the current flow in the brain to reduce the potential effects of directing stimulation to other brain sites, which suggested stimulation electrode placement at locations AF3 and CP5. Moreover, while anodal tDCS promotes increased neuronal excitability at the targeted area, the cathodal electrode promotes the opposite effect at the other site. We therefore opted to apply tACS, which is thought to promote the interaction between the stimulated areas through synchronization of oscillations (Buzsáki and Draguhn 2004; Lachaux et al. 1999; Varela et al. 2001).

Examining sensor‐level ERPs, we observed greater prospective positivity in the prospective memory in contrast with the ongoing task trials in the sham condition, consistent with previous reports (Cruz et al. 2016; Gonen‐Yaacovi and Burgess 2012; West 2011). However, neither the N300 nor frontal positivity (early and late) was detected. The N300 is associated with cue detection (Bisiacchi et al. 2011) and frontal positivity with disengagement from the ongoing task on cue detection (Wilson et al. 2013), rather than with monitoring processes. Prospective positivity, on the other hand, may be more related to strategic monitoring, given that it is thought to reflect the maintenance of current and planned tasks, as well as prospective memory retrieval (West 2011; Wilson et al. 2013).

The absence of an impact on prospective memory‐related ERP components after stimulation in the alpha‐tACS compared with the sham group suggests that other neural mechanisms underlie the prospective memory performance improvement. These components are associated with prospective memory retrieval (Gonen‐Yaacovi and Burgess 2012), suggesting that alpha‐tACS may support strategic monitoring rather than having an effect on retrieval processes. We note, however, that while prospective memory‐relevant processing may still be expected at the times and locations where prospective memory‐related ERP components have previously been reported, the absence of a sensor‐level activity difference between prospective memory and ongoing task items in the sham group means the effects of stimulation, while still potentially modulating neural processes that contribute to these components, are unlikely to be seen in amplitude modulation in sensor space. The neural processes resulting in the previously reported ERP difference between prospective memory and ongoing task items may arise from cortical processing in deeper cortical regions than can be readily detected in EEG. Neuroimaging studies have indeed identified prospective memory‐related activity in deeper cortical regions (Cona et al. 2023). The N300, for example, has been shown in a combined ERP/fMRI study to arise in dorsal PCC (O'Hare et al. 2008). We therefore performed source analyses. Furthermore, as tACS is applied at behaviorally relevant oscillatory frequencies for prospective memory, we also investigated whether stimulation resulted in persistent oscillatory power changes, given the ongoing improvement in prospective memory performance after stimulation.

The performance in the alpha‐tACS group differed during and after compared with before stimulation. In the alpha‐tACS group, differences in electrophysiological source activity in the prospective memory compared with the ongoing task condition were observed as an after‐effect over a region encompassing DLPFC and aPFC. These brain regions are thought to play a role in monitoring and attentional processes (Cona et al. 2015). Task interference on introduction of the prospective memory task, with a deterioration in ongoing task performance, is consistent with our aim to evaluate strategic monitoring, which is the approach most commonly employed for prospective memory tasks involving non‐salient, non‐focal cues (Gonen‐Yaacovi and Burgess 2012; McDaniel and Einstein 2000). Differing source activity in prospective memory and ongoing task items in the DLPFC and aPFC with alpha‐tACS, accompanied by modulation of prospective memory performance in this group, is consistent with the possibility that alpha‐tACS has an impact on prospective memory achieved through strategic monitoring.

Examining changes in oscillatory power in the alpha and theta ranges in the sham group, greater late parietal theta power was seen in prospective memory than ongoing task trials, which may at least in part reflect the processes underlying prospective positivity. The parietal lobe has an established role in memory retention/storage associated with memory processing (Li et al. 2017; Raghavachari et al. 2006). Interestingly, although we did not observe the previously reported greater parietal N300 in scalp ERPs during prospective memory than ongoing task items, theta power was greater during prospective memory than ongoing task trials at the time and location of N300. A greater N300 has been postulated to reflect prospective memory cue detection (West 2011; Cona et al. 2015), and we suggest that the greater theta power we observed reflects prospective memory retrieval processes for the prospectively encoded intention on prospective memory cue detection.

The power analyses are consistent with prospective memory performance improvement through a direct effect on monitoring processes rather than on memory retrieval processes, which subsequently led to prospective memory retrieval enhancement. When a prospective memory task was included with the ongoing task, ongoing task performance deterioration was consistent with strategic monitoring for the prospective memory task component. After stimulation in the alpha‐tACS group, lower early frontal theta power and lower alpha power were seen compared with the Sham group during ongoing task performance while a prospective memory task was maintained. An early frontal theta power reduction was reported during monitoring in an EEG study when monitoring, with externally directed attention, was encouraged (Cona et al. 2020). Prospective remembering requires ongoing interaction between attentional and mnemonic processes, and we suggest that enhancement of attention during alpha‐tACS, reflected in frontal alpha power reduction, strengthened the accompanying memory processes, leading to lower frontal theta power in this group, which persisted, together with the prospective memory performance improvement, after the stimulation was terminated. Decreased alpha power was also observed. It is possible that alpha power also decreased significantly during active stimulation with alpha‐tACS, with benefits to attention both during the active stimulation and persisting beyond. Comparing power during only prospective memory items between the alpha‐tACS and Sham groups, no differences were observed. This finding is consistent with the suggestion that alpha‐tACS did not improve prospective memory performance through direct modulation of retrieval processes only observed in prospective memory trials. While we would expect strategic monitoring processes to be present in both ongoing task and prospective memory trials, we evaluated ongoing task trials separately to avoid potential influences of prospective memory retrieval when examining monitoring. The absence of the same power differences between the alpha‐tACS and Sham groups in prospective memory as those that we detected in ongoing task items may reflect the additional prospective memory retrieval processes in the prospective memory trials. It is also possible that the number of prospective memory trials is not adequate to show differences. The low number of prospective memory trials is an important challenge in prospective memory studies. Increasing the numbers of prospective memory trials would render the task a classical dual‐task paradigm.

The absence of an ongoing task accuracy difference between blocks in the alpha‐tACS group suggests that the improved prospective memory performance was specific to the prospective memory task. However, ongoing task RTs did show a parallel improvement with prospective memory performance, with faster ongoing task RTs during and after than before stimulation. It is therefore possible that alpha‐tACS enhanced cognitive processing underpinning both task types. Attention plays a crucial role in both prospective memory and WM performance, as WM holds a subset of representations that also controls the direction of attention (Oberauer 2009, 2019) and is essential in strategic monitoring, also known as attentional monitoring, for prospective memory cues (Gonen‐Yaacovi and Burgess 2012; McDaniel and Einstein 1993; West et al. 2006). The negative impact on WM performance of introducing the prospective memory task indeed suggests that attentional resources were shifted from the ongoing task and allocated to monitoring for a prospective memory cue (Czernochowski et al. 2012; Smith 2003; Villafane Barraza et al. 2023; West and Bowry 2005). Taken together with the improvement in performance of both the prospective memory and the ongoing task components, being reflected in different behavioral measures, i.e., accuracy and RT, it is possible that different mechanisms underpin the performance improvement in prospective memory and that in ongoing task. Moreover, the electrophysiological findings point to a differential effect on prospective memory and ongoing task, with lower source activity in the left aPFC/DLPFC in the late time window in the prospective memory than in the ongoing task condition in the alpha‐tACS group.

In contrast, neither the theta‐tACS nor Sham groups showed any prospective memory performance modulation during or after stimulation compared with before. The theta‐tACS group did, however, show performance changes in the ongoing task. While accuracy did not differ during or after compared with before stimulation, RTs became faster. Indeed, previous studies have shown improvement of WM performance with theta‐tACS (Polanía et al. 2012; Röhner et al. 2018; Violante et al. 2017). These observations support the notion that the effect of stimulation on prospective memory performance is frequency‐specific, supporting the hypothesis that modulation of alpha rather than theta oscillations is key to an impact on prospective memory performance with non‐focal cues, which is likely to result from an effect on attentional networks. Increased theta power was reported during a paradigm that involved focal prospective memory cues and an ongoing task with a high working memory load and was thought to reflect working memory engagement (Cona et al. 2020). Further studies are required to evaluate potential differential effects of theta‐tACS according to prospective memory cue focality.

We expected the late time window to show changes in prospective positivity and late frontal positivity. While we observed no parieto‐occipital differences that would reflect modulation of prospective positivity, frontal areas showed lower activity during prospective memory than ongoing task trials in the alpha‐tACS group. The left aPFC plays an important role in prospective memory processing during maintenance and retrieval of an intention (Bisiacchi et al. 2011; Burgess et al. 2001, 2003; Cona et al. 2015; Momennejad and Haynes 2012). Neural correlates of intention maintenance would also be expected to be present during ongoing task as well as prospective memory trials. The difference we observed is therefore more likely to reflect prospective memory retrieval processes. Moreover, the RTs to prospective memory cues show that the responses were made at the end of this time window, so that the activity difference here is likely to reflect prospective memory retrieval. The aPFC is deemed to be involved in attentional switching (Burgess et al. 2011), so the difference between prospective memory and ongoing task trials is likely to reflect the switching that takes place only in prospective memory trials.

We observed an activity difference between prospective memory trials in the alpha‐tACS and Sham groups in the early time window, when frontal positivity and the parieto‐occipitally located N300 were expected. The difference we observed was, however, localized to the PCC. The PCC is an important part of the default mode network, and its widespread connectivity has led to the suggestion that it serves as a cortical hub (Hagmann et al. 2008). The attention to delayed intention model includes a role for the PCC in prospective memory (Cona et al. 2015, 2016, 2023). The model proposes that after prospective memory cue detection, the salience network is activated, with ventral frontoparietal areas and the PCC contributing to bottom‐up capture of externally directed attention to the prospective memory cue, and an attentional shift to the internal representation of the prospectively encoded intention. This suggestion fits with evidence suggesting that the PCC regulates attention to internally‐ and externally‐directed cognition as a part of the default mode network (Buckner et al. 2008). Any task performance is associated with a reduction in default mode network activity (Raichle 2015). We suggest alpha‐tACS may have enhanced this disengagement of the default mode network, supporting attentional capture by the prospective memory cue. The default mode network, and particularly the PCC, have previously been linked to internal distraction, as they play a role in task‐irrelevant processes such as mind wandering (Christoff et al. 2009; Fox et al. 2015). During tasks requiring externally oriented attention, the PCC is typically deactivated, and failure to suppress it adequately is associated with attention lapses and poorer task performance (Weissman et al. 2006; Wen et al. 2013). Fluctuations in PCC activity have indeed been proposed to serve as a quantitative neural measure of internal distraction, as individuals with higher WM capacity experienced lower internal distraction through more robust suppression of PCC activity (Rajan et al. 2019). In the case of prospective memory, we suggest that the PCC deactivation supports strategic monitoring, maintaining attention to the prospective memory task and facilitating task switching from the ongoing task to the prospective memory response. This interpretation fits with our findings of lower PCC activity during prospective memory trials in the alpha‐tACS than in the sham group and greater prospective memory accuracy in the alpha‐tACS group during and after than before stimulation.

We consider several avenues for future extensions of this work as well as several potential limitations of the current study. The first consideration is that the EEG data were recorded during task performance after the stimulation. This approach focused on the electrophysiological after‐effects of stimulation. While this focus meant that stimulation artifacts were not a consideration, with application of artifact removal methods, future work could include evaluation of the online effects of stimulation on electrophysiological correlates of prospective memory as well as behavioral effects. It is also important to note that the study was based on a prospective memory paradigm using non‐focal prospective memory cues, increasing the likelihood that participants employed a strategic monitoring approach. Future work, with paradigms involving focal cues to encourage greater use of spontaneous retrieval, is required, as well as tasks involving time‐based prospective memory, to give a more complete picture of the impact of tACS on prospective memory more generally. We also highlight the consideration of the limited spatial resolution provided by EEG‐based source analyses. Future work should include high‐resolution functional imaging to investigate whether the task‐related activations and changes following tACS are also observed in the brain areas identified here. We also consider the interpretation of the mechanism by which alpha‐tACS might support prospective memory. While entrainment of oscillations at the stimulation frequency is proposed to facilitate communication between brain areas engaged in a task, EEG recordings would be required to be made in future work during the stimulation and task performance to evaluate this possibility. Given our data recording after stimulation, we can only report on potential after‐effects of the stimulation. A further potential limitation is that although the stimulation groups did not differ in age, KAI scores, or baseline WM performance, a between‐group behavioral comparison could not be made due to differing pre‐stimulation WM performance between the groups. We nonetheless compared EEG activity in the alpha‐tACS group during prospective memory with that in the Sham group, based on the within‐subject behavioral modulation that we observed on alpha‐tACS application. This comparison enabled evaluation of whether the prospective memory behavioral performance change on alpha‐tACS application was specific to alpha‐tACS or reflected a practice effect also observable in the Sham group. The finding of a difference between the two groups suggests that a prospective memory‐specific modulation was achieved in the alpha‐tACS group. The approach was made possible by the examination of established neural correlates of prospective memory. Future work with larger participant numbers should yield comparable WM baseline performance across groups, enabling direct comparison of behavioral effects between stimulation groups. Finally, while stimulation was applied to the DLPFC, we observed neural modulation in the aPFC. The findings may reflect network effects, as both these regions are established as being engaged in prospective memory. However, given the large stimulating electrodes used, we cannot exclude stimulation application to both these adjacent areas. Future studies applying focal tACS in combination with high‐resolution functional imaging may enable a differentiation between stimulating aPFC and DLPFC and allow separation of their specific roles in prospective memory.

## 5 Conclusions

Modulation of prospective memory performance and its neural correlates through alpha‐tACS provides evidence for a role for alpha oscillations in prospective memory processing, which is likely to reflect attentional processes underpinning strategic monitoring.

## Author Contributions

**Bruno de Matos Mansur:** conceptualization, data curation, formal analysis, investigation, methodology, software, validation, visualization, writing – original draft, writing – review and editing. **Viviana Villafane Barraza:** data curation, investigation, writing – review and editing. **Angela Voegtle:** data curation, investigation, writing – review and editing. **Christoph Reichert:** conceptualization, methodology, supervision, writing – review and editing. **Slawomir J. Nasuto:** conceptualization, investigation, methodology, supervision, validation, writing – original draft, writing – review and editing. **Catherine M. Sweeney‐Reed:** conceptualization, data curation, formal analysis, funding acquisition, investigation, methodology, project administration, resources, supervision, validation, visualization, writing – original draft, writing – review and editing.

## Ethics Statement

The Local Ethics Committee of the Otto von Guericke University Magdeburg granted ethical approval. All participants provided informed, written consent before study inclusion, in accordance with the Declaration of Helsinki, and were informed of their right to cease participation at any time without providing reasons.

## Conflicts of Interest

The authors declare no conflicts of interest.

## Data Availability

The scripts used for data analyses are freely available from mne and fieldtrip websites, and the anonymous data for generating the figures are available on Figshare (https://figshare.com/s/5f3624e119ddcb45e624). The data that support the findings of this study are available from the corresponding author upon reasonable request.
